# Caregiver Burden in Epilepsy: Determinants and Impact

**DOI:** 10.1155/2014/808421

**Published:** 2014-04-08

**Authors:** Ioannis Karakis, Andrew J. Cole, Georgia D. Montouris, Marta San Luciano, Kimford J. Meador, Charitomeni Piperidou

**Affiliations:** ^1^Department of Neurology, Emory University School of Medicine, Atlanta, GA, USA; ^2^MGH Epilepsy Service, Massachusetts General Hospital, Harvard Medical School, Boston, MA, USA; ^3^Department of Neurology, Boston Medical Center, Boston University School of Medicine, Boston, MA, USA; ^4^Department of Neurology, University California San Francisco, San Francisco, CA, USA; ^5^Department of Neurology, Stanford School of Medicine, Stanford, CA, USA; ^6^Department of Neurology, Democritus University of Thrace, Alexandroupolis, Greece

## Abstract

*Aim*. Caregiver burden (CB) in epilepsy constitutes an understudied area. Here we attempt to identify the magnitude of this burden, the factors associated with it, and its impact to caregiver quality of life (QOL). *Methods*. 48 persons with epilepsy (PWE) underwent video-EEG monitoring and their caregivers completed questionnaires providing demographic, disease-related, psychiatric, cognitive, sleep, QOL, and burden information. *Results*. On regression analysis, higher number of antiepileptic drugs, poorer patient neuropsychological performance, lower patient QOL score, and lower caregiver education level were associated with higher CB. Time allocated to patient care approximated but did not attain statistical significance. A moderate inverse correlation between CB and caregiver QOL physical component summary score and a stronger inverse correlation between CB and caregiver QOL mental component summary score were seen. *Conclusion*. In a selected cohort of PWE undergoing video-EEG monitoring, we identified modest degree of CB, comparable to that reported in the literature for other chronic neurological conditions. It is associated with specific patient and caregiver characteristics and has a negative effect on caregiver QOL.

## 1. Introduction

Epilepsy is an unpredictable, often chronic and debilitating disorder that impacts not only those bearing with it but also those who care for them. Epilepsy is thought to affect more than 100 million individuals and their families worldwide at some point of their lives, thus constituting a major, universal, public health issue [1].

It is well established that epilepsy impacts the quality of life (QOL) of patients. Loss of control and independence, low self-esteem, fear, depression, stigmatization, lifestyle, social and employment restrictions, and financial strains are ways in which this impact occurs [2]. The same factors also indirectly affect care providers for those patients.

**Figure 1:**
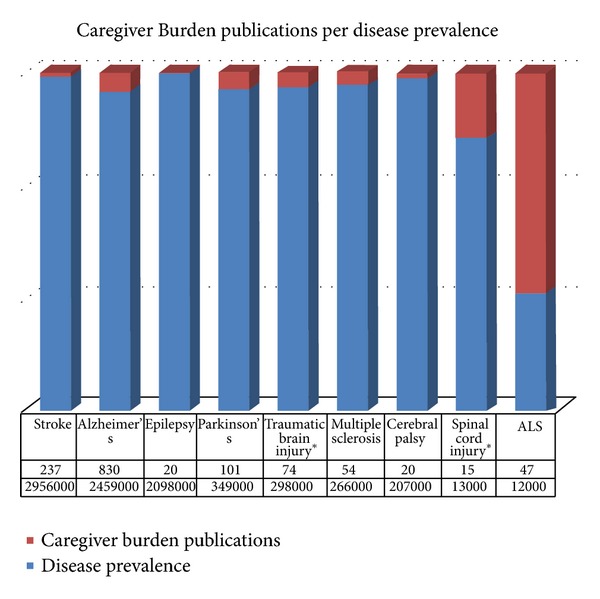
Publications on caregiver burden (pubmed search, accessed April 2013) for various neurologic disorders in proportion to disease prevalence (incidence for disease with *) [48].

In contrast to other chronic medical conditions such as congestive heart failure [3], chronic obstructive pulmonary disease [4], chronic renal failure [5], cancer [6], and chronic neurological disorders such as stroke [7], Alzheimer's disease [8], Parkinson's disease [9], multiple sclerosis [10], amyotrophic lateral sclerosis [11], traumatic brain [12], or spinal cord injury [13], the impact of epilepsy on the family constitutes an understudied area. As illustrated in Figure 1, despite being the fourth most common neurological condition, caregiver burden in epilepsy has attracted disproportionally less attention than in less prevalent neurological conditions such as Alzheimer's disease, multiple sclerosis, Parkinson's disease, and amyotrophic lateral sclerosis. When caregiver burden and QOL-related issues have been explored, most studies have focused on the pediatric population [2, 14–28]. The data on caregivers of adult patients remains sparse [29–35] and most studies have been performed outside the United States.

Given the scarcity in the literature in this area, we sought to quantify caregiver burden in epilepsy, determine the relative contributions of patient- and caregiver-related factors, and ascertain the impact that this burden has to the caregiver health-related QOL. We also identify implications of our findings and future directions in the field of caregiver burden and QOL in epilepsy both from clinical and research standpoints.

## 2. Methods

### 2.1. Participants

This is a cross-sectional study conducted between September 2009 and June 2011 at Massachusetts General Hospital (MGH). Adult patients admitted electively to the Epilepsy Monitoring Unit (EMU) for continuous video-EEG monitoring were asked to participate by completing a series of questionnaires and undergoing bed-side cognitive evaluation. Patients who were non-English speakers or unable to read and write due to cognitive impairment were excluded. Caregivers who accompanied them were also asked to complete questionnaires. Caregiver was defined as the family member who was primarily responsible for providing every-day care for the patient. After the monitoring was completed, only the patients with documented epileptic seizures whose caregivers completed their questionnaires were included in the analysis, while patients without a caregiver participant or patients with nonepileptic seizures, mixed disorder, or unclear diagnosis were excluded along with their caregivers. Out of 190 admissions during this study period, 14 were invasive recordings where the anesthesia/postoperative state of the patients may have interfered with their ability to reliably answer all surveys administered and another 12 admissions were repeated admissions. The total number of admitted available patients was therefore 164, out of which 126 were elected to participate leading to responder's rate of approximately 77%. From those, 80 were proven to have epilepsy. The remaining 46 patients were diagnosed with psychogenic or other non epileptic events, mixed epileptic and nonepileptic events or had no events recorded during their stay. 48 of the 80 persons with epilepsy (PWE) had a caregiver escorting them to the EMU and those 48 patient-caregiver pairs comprised the final study population. Consent for participation was obtained from all eligible available caregivers. More male patients had an available caregiver present. Otherwise, PWE with an available caregiver did not differ significantly compared to those without one. That recruitment process yielded 48 PWE-caregiver pairs which was the focus of the study. The study was approved by the institutional review board.

### 2.2. Questionnaires and Procedures

Participating patients completed questionnaires providing demographic (age, gender, race, religion, employment, education, living situation, and marital status) and epilepsy-related (age of epilepsy onset, epilepsy duration in years, average number of seizures per month in the past year, number of AED, and self-reported compliance) information. The information collected was cross-validated with medical records review.

Anxiety and depression levels were measured using the Beck anxiety [36] and Beck depression [37] inventories, respectively. Those are 21-item inventories that assess the presence and degree of affective, cognitive, motivational, and psychomotor components. Each item is scored from 0 to 3 and the aggregate score is 0–63. Higher scores depict higher levels of psychopathology (depression: 1–10: normal, 11–16: mild depression, 17–20: borderline depression, 21–30: moderate depression, 31–40: severe depression, and >41: extreme depression; anxiety: 0–21: very low anxiety, 22–35: moderate anxiety, and >36: high anxiety). Both have been extensively used previously in epilepsy research [38]. Sleep quality was assessed by completing the Epworth sleepiness scale [39] and the sleep apnea section of the sleep disorder questionnaire (SDQ-SA) [40]. The Epworth sleepiness scale is a brief questionnaire rating the chances that they would doze off or fall asleep when in eight different situations commonly encountered in daily life. A score of 0–3 is given to each situation and the aggregate score is 0–24. Higher scores are suggestive of higher sleepiness level (a cutoff of >10 is generally interpreted as daytime sleepiness) [39]. While not specific to patients with epilepsy, it has been widely used to assess sleepiness in a host of diseases including epilepsy [41]. The SDQ-SA has also been commonly applied to the epilepsy population [42]. A score equal to or more than 36 for men and 32 for women is considered to have approximately 80% sensitivity and specificity for polysomnographically proven sleep apnea [40]. QOL was evaluated by completing the QOLIE-31 instrument. QOLIE-31 is one of the most commonly applied QOL instruments in epilepsy with good reliability and validity [43]. The 31-item self-administered questionnaire has seven subscales: seizure worry, overall QOL, emotional well-being, energy/fatigue, cognitive function, medication effects, and social functioning. A score ranging from 1 to 100 is obtained from each subscale with higher scores indicating better QOL. Cognitive evaluation was performed by a neurologist via administration of the Montreal Cognitive Assessment (MoCA) test [44]. This is a brief screening tool that has been shown to be superior to the commonly used mini-mental status examination for the detection of mild cognitive impairment in the epilepsy population [45]. By assessing multiple cognitive functions (visuospatial/executive, naming, memory, attention, language, abstraction, delayed recall, and orientation) an aggregate score of 0–30 is created. Higher scores are associated with better cognitive state (a cutoff of <26 is considered abnormal). All these evaluations took place on the day of the admission under electrographic guidance to ensure the absence of subclinical electrographic seizure activity affecting some of the responses. At the time of the testing, the patients were maintained on their home AED(s) and had not been yet sleep deprived with the intent that their answers would be representative of their baseline state in the ambulatory setting.

Caregivers accompanying the patients also completed questionnaires providing demographic information (age, gender, race, religion, employment, education, marital status, cohabitation, and time spent for patient care in hours per week). The latter was loosely defined as the time devoted to everyday activities where caregiver participation was indispensable including AED provision, outpatient and emergency department visits, and driving for any patient-related activity. Given the lack of a disease-specific questionnaire to assess their burden, the Zarit caregiver burden inventory was used. This is a 22-item inventory derived from the original 29-item inventory [46]. It is the most widely used standardized, validated scale to assess caregiver burden, administered previously in various neurological disorders, including epilepsy [14, 34]. The 22 items evaluate the effect of disease on the caregiver's QOL, psychological suffering, financial difficulty, shame, guilt, and difficulty in social and family relationships. Scores range from 0 to 88 with higher scores indicating higher burden (<20: little or no burden, 21–40: mild-to-moderate burden, 41–60: moderate-to severe burden, 61–88: severe burden). Their health-related QOL was assessed by administering the second version of the SF-36 generic questionnaire (SF36v2) [47]. This is a generic QOL instrument that assesses eight health concepts (physical functioning, role limitation caused by physical problems, bodily pain, general health perception, vitality, social functioning, role limitation caused by emotional problems, and mental health). Scores standardized to norms and weighted averages are used to create a physical component summary (PCS) and a mental component summary (MCS) composed by the first and last four of the aforementioned health concepts, respectively. All health dimension scores are standardized to normal by employing a linear transformation of data originally scored on a 0–100 scale Norm-based scores have a mean of 50 and a standard deviation of 10 in the general US population. Therefore, any score <50 for any health dimension and component scale falls below the general population mean and each point represents 1/10 of a standard deviation. This allows direct comparison among different populations [47] and has established precedence in epilepsy caregiver research.

Various paraclinical (e.g., laboratory, electroencephalographic and radiological) data were collected as part of standard of care. Routine AED levels were drawn on admission prior to initiation of gradual withdrawal. For patients on more than one AED, they were deemed to be above, within, or below the antiepileptic drug reference range of their regimen depending on the serum level of the majority of drugs in their regimen. EEG data pertained to the initial recording during the completion of the questionnaires (normal, slow, epileptiform) including the maximal posterior dominant rhythm at the time of completion and the final epilepsy monitoring unit report for classification of their seizure type (partial with or without secondary generalization and primarily generalized), epilepsy type (unitemporal right or left, bitemporal, extratemporal right or left, multilobar or idiopathic generalized epilepsy), and etiology (symptomatic, cryptogenic, or idiopathic). Radiological data included findings of the last patient's brain magnetic resonance imaging (normal, mesial temporal sclerosis, diffuse atrophy, vascular, developmental, or other abnormality) obtained before, during, or right after this monitoring.

### 2.3. Analysis

Summary scores were created for all the aforementioned variables and descriptive statistics were used. Univariate associations between the Zarit burden score as the outcome of interest and the various patient and caregiver related predictors were explored by using *t*-test or one-way ANOVA and Pearson correlation or nonparametric equivalents when appropriate. Statistical significance was set at 0.05. Those variables identified as statistically significant in the univariate analysis were subsequently fitted in a multivariate linear regression model in order to conduct an adjusted evaluation of associated factors of caregiver burden. Finally, Pearson correlation coefficient was used to investigate the association between the caregiver burden score and each of the caregiver QOL scale score. Statistical analysis was performed in SAS 9.3 (North Carolina) and STATA 11 (College Station, TX).

## 3. Results

Demographics are detailed in Table 1. The mean age of the patients was 36 years. The majority of the patients were men, Caucasian, and had obtained higher education. Nearly half were married and two-thirds were employed. Patients had epilepsy for approximately 16 years, averaging 4 seizures per month, mainly partial with secondary generalization and taking on average 2 AED. The majority had symptomatic temporal lobe epilepsy. Their AED levels on admission were mostly in the reference range and their average score on the MoCA assessment of cognitive function was 25. Their average depression score was nearly 11, and anxiety score was 13. Mean Epworth sleepiness scale score was 8 and mean SDQ-SA score was approximately 25. The overall QOLIE-31 score was nearly 56.

The mean age of the caregivers was 46. Most were Caucasian women, married, employed, of higher education, and cohabitated with the patients they cared for. Their average Zarit burden score was 20, that is, on the cusp of mild-to-moderate range, overall comparable with other chronic neurological conditions where the same burden questionnaire was applied (Table 2). The physical component scale of their QOL score averaged 54 points, while the mental component scale averaged 45 points.

In the univariate analysis, higher AED number, lower patient's neuropsychological scores, lower scores in many of the subscales of patient's QOL scale (i.e., seizure worry, emotional well-being, cognitive functioning, and social functioning) including the overall score as well as lower caregiver education level, and increase in the time spent with the patient were shown to be associated with higher disease burden to the caregiver (Table 3). In the multivariate analysis, the same factors of caregiver burden were confirmed but time allocated to patient care approximated but did not retain statistical significance (Table 4).

**Figure 2:**
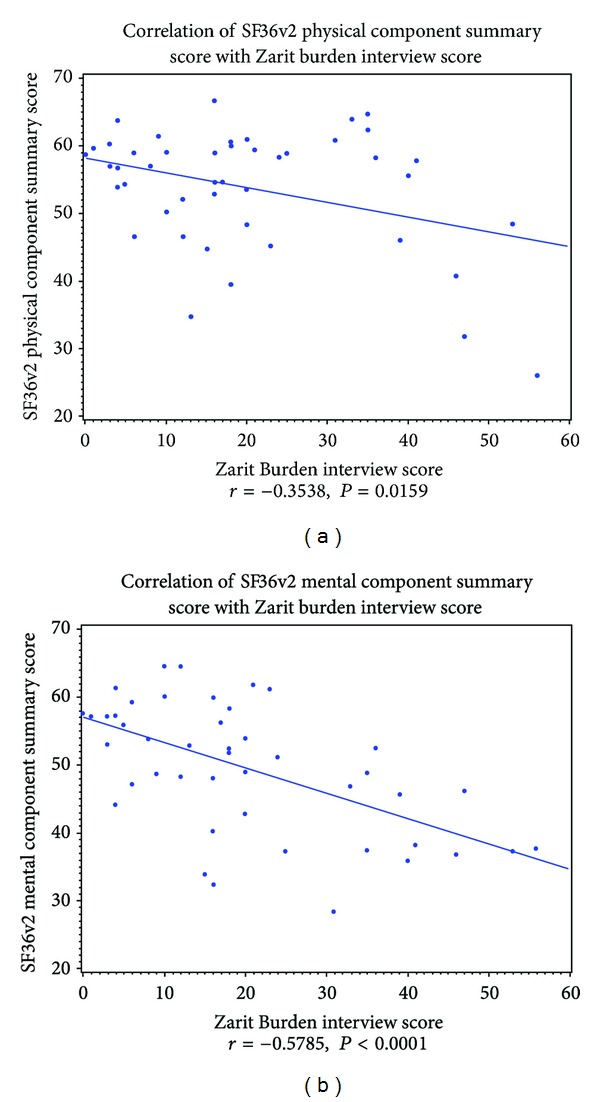
Correlation between caregiver burden and each of the components of caregiver quality of life (i.e., physical component scale (PCS) and mental component scale (MCS)).

There were a statistically significant moderate inverse correlation between caregiver burden and caregiver QOL physical component summary score (*r* = −0.35, *P* = 0.01) and a stronger inverse correlation between caregiver burden and caregiver QOL mental component summary score (*r* = −0.57, *P* ≤ 0.0001) (Figure 2).

## 4. Discussion

In this selected cohort of PWE undergoing video-telemetry and their caregivers, we identified the following: (a) epilepsy is associated with modest degree of burden to the caregiver, which is overall comparable to burden from other chronic neurologic conditions reported in the literature; (b) the number of AED, the patient's neuropsychological state, the patient's quality of life, and caregiver education are associated with caregiver burden; and (c) caregiver burden has a negative impact on caregiver health-related quality of life.

As illustrated in Table 2, regardless of differences in the pathophysiology of other neurological disorders and methodological variability in their research, the identified magnitude of caregiver burden in epilepsy in our study is overall comparable to other neurological conditions where similar instruments were administered, including stroke [7], Alzheimer's disease [8], Parkinson's disease [9], multiple sclerosis [10], amyotrophic lateral sclerosis [11], and traumatic brain [12] or spinal cord injury [13]. In addition to the chronicity seen in those neurological conditions, epilepsy can often start much earlier in life; it is characterized by a paroxysmal course that introduces the unique strain of unpredictability and it is related to high grade of stigmatization. Also, caregiver QOL scores in other neurological conditions do not deviate significantly from what is reported here for epilepsy, when similar scales were used. This further underscores the aforementioned disparity between caregiver research in the 4th most common neurological condition (past migraine, stroke, and Alzheimer) [48] compared to less prevalent diseases.

Our prior knowledge of the caregiver burden in epilepsy and its associated effect on caregiver QOL is deficient. Most extant studies have focused on the pediatric population. In the adult population, most studies have been performed in the outpatient setting and outside the United States. In particular, outpatient studies performed in the Netherlands identified a trend of decreased mental component of QOL in caregivers of refractory patients [33]. No specific patient or disease characteristic appeared to drive caregiver QOL [33]. On the contrary, caregiver self-perceived burden of care [33] and coping style [32] were deemed to be more reliable indicators. Using a control group for comparison, a study of 257 caregivers escorting patients to outpatient clinics in Sudan revealed lower QOL scores for caregivers who were children of the patients, female, and had lower education attainment [35]. Another study of 231 caregivers of patients attending an outpatient clinic in Nigeria identified a median Zarit burden score of 25 [30]. Higher burden was associated with younger patient's age, patient's unemployment, longer disease duration, shorter periods of seizure freedom, family history of epilepsy, and rural residence, possibly accounting for poorer access to health care [30]. In Brazil, Westphal-Guitti et al. compared 50 adolescent and adult patients with juvenile myoclonic epilepsy (JME) and another 50 with temporal lobe epilepsy (TLE) along with their caregivers [34]. Mild-moderate caregiver burden, averaging 22 for JME and 30 for TLE in the Zarit scale, was identified. For JME patients that burden correlated with poorer emotional, social, and physical domains of the caregivers' QOL measured with SF-36, while for TLE patients the emotional component was primarily affected [34]. Another study of 65 patient-caregiver pairs from Hong Kong identified below average scores on the QOL measure applied and severe levels of depression and anxiety in 14% and 22% of caregivers, respectively [29]. The authors indicated that seizure severity and age at onset are negatively correlated with psychosocial adjustment of caregivers; on the other hand, perceived support level had a positive impact in their well-being and QOL [29]. Earlier exploratory investigation of 44 families living with epilepsy in the United Kingdom suggested increased levels of anxiety and depression in caregivers of patients with severe drop attacks and history of status epilepticus [31]. Social dissatisfaction and low levels of support were again voiced as major concerns by the caregivers [31].

Our findings partially concur with the preexisting literature. Similar to Westphal-Guitti et al. [34] and Tajudeen Nuhu et al. [30], we were also able to identify burden related to the care of patients with epilepsy, yet relatively milder than previously reported. In agreement with the Brazilian [34] and the Dutch studies [33], we also recognized heavier impact in the mental component of caregiver QOL. The variability of burden magnitude and predictors reported in the literature including our study probably accounts for the broad difference in study populations, the multifaceted nature of epilepsy, and the variable research methodology applied.

There are certain advantages to our study. The focus was on adult patients, where most of the literature is sparse, who could complete the surveys independently. That prevented potential bias inevitably incurred by proxy-reports in the pediatric caregiver literature [49]. The patients recruited had well-defined epilepsy proven with inpatient video-EEG monitoring. That excluded potential misclassification that may inadvertently occur when such methods are applied in the outpatient setting. We monitored and minimized factors that may have interfered with patient's testing such as seizures or commonly applied procedures in the EMU (e.g., antiepileptic medication withdrawal or sleep deprivation). Cross-reference with medical records provided an additional checkpoint for accuracy. The data collected were thorough and covered most of the parameters reported to be associated with health-related QOL in epilepsy, including paraclinical data such as AED levels, an understudied field previously. Thus, multiple patient- and caregiver-related factors were taken into account when assessing caregiver burden.

On the other hand, there are limitations to acknowledge. First, self-reporting nature of the study bears a risk of recall bias. Yet, self-report scales are widely used, cost-effective methods for both diagnostic assessment and for outcome evaluation. Admittedly though they are not as exhaustive and objective as standardized cognitive and psychiatric interviews or physiologic sleep recording procedures. Second, the modest sample size of caregiver participants may have underpowered our study for the detection of additional associations. Third, despite the extensive evaluation of patient-associated factors, caregiver-related aspects that may have been associated with their burden, such as social support, financial information, comorbidities, and depression and anxiety scales, were not directly addressed. They constitute, however, components of the Zarit burden inventory used. Fourth, the cross-sectional nature of the study prevented further insight into the evolution of these associations longitudinally as well as inference of causation. Fifth, we restricted our analysis to PWE who were accompanied by caregivers who completed their questionnaires. Although the patients who were not escorted by caregivers did not differ substantially from those who did, our study sample may still not be fully representative of the caregiver population for PWE. Similarly, the study population was mostly in families of higher socioeconomic and educational status. They were recruited in the EMU of a tertiary referral center of a US hospital. While this recruitment strategy allowed rigorous characterization of their epilepsy, QOL, and burden associations, it may have significantly limited generalizability of our findings to the community and to other countries where different socioeconomic barriers exist. The hospitalization itself for further epilepsy evaluation and treatment may have inadvertently affected some of the burden and QOL scores that both PWE and their caregivers provided. Finally, the absence of a nonepilepsy patient-caregiver control group limited our ability to directly compare our findings with other chronic neurologic or medical disorders in which caregivers also play a significant role.

The findings of this study have potential implications both for clinical practice and research paradigms. In clinical practice, physicians should consider incorporating the caregiver into their assessment and treatment plan in an effort to eventually improve the patient's quality of life. Caregiver counseling and education, evaluation and treatment of evolving caregiver psychopathology, and individualized and/or group multidisciplinary interventions to provide physical, emotional, social, and financial support to the caregiver may ameliorate caregiver burden. This may in turn provide significant reciprocal benefit to the QOL of the patient which appears to be inextricably interwoven as shown in our study. Previous studies on caregivers of patients with dementia have corroborated that potential [50]. Further, advocacy groups should include caregiver feelings and needs into their agenda and expert opinion panel reviews as well as national clinical guidelines should further emphasize caregiver QOL as one of the core quality measures in the evaluation and management of epilepsy [51]. In the research field, the focus of investigation should expand to incorporate the family well-being. Our findings suggest associations that warrant further examination in future studies and especially in broader socioeconomic settings in order to elucidate further both the predictors as well as the influence of caregiver burden to their QOL and ultimately to the patient's QOL. Epilepsy specific QOL measures need to be created and validated for the caregivers of PWE and incorporated into future medication and intervention related clinical trials in epilepsy. As also underscored by the recently published Institute of Medicine report on epilepsy, there is need for rigorous research in this understudied field [52], and funding agencies should consider this important issue.

## 5. Conclusion

In a selected cohort of persons with epilepsy undergoing video-EEG monitoring, we identified modest caregiver burden. This burden is comparable to that reported in the literature for other less prevalent, chronic neurological conditions, although it has been under investigated, particularly for the adult epilepsy population. It appears to be associated with three patient-related factors (i.e., AED number, cognitive performance, and quality of life) and one caregiver-related factor (i.e., education attainment). This burden places a toll to the stakeholders of epilepsy care both for their physical and even more for their psychological well-being. These findings call for further investigation of caregiver burden and quality of life in epilepsy in broader socioeconomic settings and for their inclusion in the physicians' treatment plan and epilepsy care quality measures.

## Figures and Tables

**Table:** (a) Patient characteristics

	Epilepsy patients *N* = 48
Demographic characteristics	
Age (mean ± SD)	36.52 ± 12.47
Gender (*n*, % female)	28 (58.33%)
Race (*n*, % caucasian)	45 (93.75%)
Religion (*n*, % Christian)	38 (80.85%)
Employment (*n*, % employed)	32 (66.67%)
Education (*n*, % some college and beyond)	38 (79.17%)
Living situation (*n*, % living with family or others)	44 (91.67%)
Marital status (*n*, % married)	23 (47.92%)

Epilepsy characteristics	
Age of onset of epilepsy (mean ± SD)	19.75 ± 14.71
Duration of epilepsy in years (mean ± SD)	16.05 ± 13.58
Number of seizures per month (median, IQR)	4 (6)
Number of AED (median, IQR)	2 (2)
Compliance (*n*, % compliant)	39 (84.78%)
Type of seizures	
Partial without generalization	12 (25%)
Primarily generalized	4 (8.3%)
Partial with secondary generalization	32 (66.67%)
Etiology	
Symptomatic	31 (64.58%)
Cryptogenic	13 (27.08%)
Idiopathic	4 (8.33%)

Paraclinical characteristics	
AEDs level	
Within reference range	27 (75%)
Below reference range	5 (13.89%)
Above reference range	4 (11.11%)
EEG posterior dominant rhythm	9.43 ± 1.15
EEG findings	
Slowing	6 (12.77%)
Interictal spikes	23 (48.94%)
Normal	18 (38.30%)
EMU diagnosis	
Left TLE	14 (29.17%)
Right TLE	13 (27.08%)
Bitemporal	2 (4.17%)
Left extra-TLE	8 (16.67%)
Right extra-TLE	2 (4.17%)
Multilobar	5 (10.42%)
IGE	4 (8.33%)
MRI Findings (*n*, % abnormal)	33 (68.75%)

Neuropsychological and sleep characteristics	
Montreal Cognitive Assessment Score (MoCA)	25 ± 4.22
Beck Depression Inventory	10.93 ± 8.65
Beck Anxiety Inventory	13.02 ± 11.08
Epworth Sleepiness Scale	8.19 ± 4.19
Sleep disordered questionnaire for sleep apnea (SDQ-SA)	24.70 ± 8.91

Quality of life characteristics (QOLIE-31)	
Seizure worry	48.53 ± 30.23
Overall quality of life	61.68 ± 22.27
Emotional Wellbeing	64.57 ± 20.94
Energy/Fatigue	46.46 ± 22.42
Cognitive Functioning	55.35 ± 25.76
Medication Effects	49.09 ± 25.86
Social Functioning	51.60 ± 29.69
Overall Score	55.98 ± 18.44

SD: standard deviation, IQR: inter-quartile range, AEDs: antiepileptic drugs, EMU: epilepsy monitoring unit, EEG: electroencephalogram, TLE: temporal lobe epilepsy, IGE: idiopathic generalized epilepsy, MRI: magnetic resonance imaging, QOLIE-31: Quality of Life 31 questionnaire.

**Table:** (b) Caregiver characteristics

	Caregivers *N* = 48
Demographic characteristics	
Age (mean ± SD)	46.18 ± 13.20
Gender (*n*, % female)	33 (68.75%)
Race (*n*, % caucasian)	45 (93.75%)
Religion (*n*, % Christian)	36 (75%)
Relationship to patient (*n*, %)	
Spouse/partner	28 (58.34%)
Parent/sibling	18 (37.50%)
Other	2 (4.17%)
Employment (*n*, % employed)	34 (70.83%)
Education (*n*, % some college and beyond)	39 (81.25%)
Marital status (*n*, % married)	38 (79.17%)
Cohabitation with patient (*n*, %)	43 (89.58%)
Time spent for patient care (hours) per week	11.43 ± 21.22

Quality of life characteristics (SF36v2)	
Physical Component Summary (PCS)	53.91 ± 8.86
Mental Component Summary (MCS)	45.51 ± 11.31

Burden characteristics	
Zarit Burden Inventory	20.02 ± 14.47

SD: standard deviation, SF36v2: short form 36 health survey version 2.

**Table 2:** Caregiver burden in epilepsy compared to other chronic neurological conditions.

Author/year	Disease	Caregivers number	Zarit burden interview mean score
Carod-Artal et al., 2009 [7]	Stroke	200	27.2
Schölzel-Dorenbos et al., 2009 [8]	Alzheimer's disease	97	12.8
Martínez-Martín et al., 2007 [9]	Parkinson's disease	79	26.5
Rivera-Navarro et al., 2009 [10]	Multiple sclerosis	278	22
Pagnini et al., 2011 [11]	Amyotrophic lateral sclerosis	37	19.5
Bayen et al., 2013 [12]	Traumatic brain injury	66	25.1
Current study	Epilepsy	48	20

**Table:** (a) Patient characteristics associated with caregiver burden

Variable	*P* value
Demographic characteristics	
Patient age	0.79
Patient gender	0.77
Patient race	0.62
Patient religion	0.85
Patient employment	0.48
Patient education	0.83
Living situation	0.07
Marital status	0.76

Epilepsy characteristics	
Age of onset epilepsy	0.36
Duration of epilepsy	0.16
Number of seizures per month	0.89
Number of AEDs	**0.0009 **(**r** = 0.46)
Compliance	0.40
Type of seizures	0.78
Etiology	0.52

Paraclinical characteristics	
AEDs level	0.70
EEG posterior dominant rhythm	0.58
EEG findings	0.95
EMU diagnosis	0.51
MRI findings	0.10

Neuropsychological and sleep characteristics	
Patient MoCA	**0.003 **(**r** = −0.41)
Patient Beck Depression	0.18
Patient Beck Anxiety	0.10
Patient Epworth	0.11
Patient SDQ-SA	0.84

Quality of life characteristics (QOLIE-31)	
Seizure worry	**0.005 **(**r** = −0.39)
Overall quality of life	0.07
Emotional well-being	**0.04 **(**r** = −0.30)
Energy/Fatigue	0.4413
Cognitive functioning	**0.006 **(**r** = −0.39)
Medication effects	0.32
Social Functioning	**0.05**
Overall score	**0.004 **(**r** = −0.40)

**Table:** (b) Caregiver characteristics associated with caregiver burden

Variable	*P*-value
Age	0.15
Gender	0.50
Race	0.62
Religion	0.44
Relationship to patient	0.16
Employment	0.94
Education	**0.05 **(**r** = −0.27)
Marital status	0.60
Cohabitation	0.44
Time spent for patient care	**0.01 **(**r** = 0.37)

**Table 4:** Factors associated with caregiver burden: multivariate analysis.

Variable	Beta coefficient	Standard error	*P* value
Number of AEDs	5.14	2.03	**0.01**
Patient MoCA	−0.78	0.38	**0.05**
QOLIE-31 overall score	−0.22	0.09	**0.02**
Caregiver education	−11.76	3.98	**0.005**
Time spent for patient care	0.15	0.08	0.06
